# Charles J. Essig, M.D., D.D.S.

**Published:** 1902-01

**Authors:** 


### Obituary.

### CHARLES J. ESSIG, M.D., D.D.S.

DIED at Wallingford, Pa., his suburban residence, December 2, 1901, Dr. Charles J. Essig, after a few days' illness of pneumonia.

Dr. Essig was born in Philadelphia July 23, 1841, and continued through his sixty years of life a resident and practitioner of dentistry in that city.

His father was Christian S. Essig, and his grandfather was an officer in the Holland naval service.

His early education was acquired in the public schools of Philadelphia. After graduation he entered an attorney's office for the study of law, but this not being to his taste he was finally, in his seventeenth year, September 10, 1857, placed with William R. Hall, who at that time enjoyed the deserved reputation of being the best porcelain block manufacturer in Philadelphia, if not in the country. At that period very few dental practitioners of reputation would use, upon a gold base, any other but block teeth and those made for each individual case. This continued until the general adoption of rubber bases about 1858, which had the effect of destroying much of the skilled mechanical dentistry of that time and that of the years antedating it. Dr. Essig entered, therefore, into dental studies at a transition period. And it seemed, at the time, as though the skill acquired would be lost through the entire change of methods. It subsequently proved the best foundation possible for his future life work. In the Hall laboratory were constructed all the usual mechanical appliances for the oral cavity used in dentistry. Into this valuable experience Dr. Essig was inducted at the most plastic period of life. Fortunately for him he possessed in large degree the mechanical taste that could receive the full benefit from such an experience.

He remained in the Hall Laboratory until his majority, a period of four years. Immediately subsequent to this, 1862, he fitted up a laboratory of his own on Arch Street below Seventh, and at a later date removed to Tenth and Arch Streets.

About the year 1867 he abandoned the preparation of mechanical work for dentists and established a dental supply house in

Baltimore, Md. This venture did not prove satisfactory and was abandoned after a comparatively brief period.

He was married in 1868 to Miss Mary Sturges, daughter of George Sturges.

Returning from Baltimore to Philadelphia in 1869, he entered into copartnership in 1870 with Dr. Louis Jack in the prosthetic portion of the latter's practice.

In 1872, when Dr. Jack removed to Sixteenth and Locust Streets, Dr. Essig became associated with him in operative dental practice. This association terminated by mutual consent in 1875, but he still retained offices in the building. Through these favorable conditions, coupled with his own skill, he gradually built up a large practice from the best elements in the city.

It is greatly to the credit of both these prominent operators that after all their years of associated effort, Dr. Jack could write of this period that "notwithstanding his (Essig's) strong nature and sensitiveness, our intercourse through all these years had been harmoniously sustained."

It was subsequently to his return from Baltimore that Dr. Essig matriculated at Jefferson Medical College, and graduated in 1871. His motives in taking the medical degree in preference to the dental may only be surmised, but it is presumed that he regarded his practical knowledge of dentistry as fully equal to the dental degree of that period.

Before his graduation in medicine, he spent much time in the chemical laboratory conducted by Dr. Henry Leffmann, not being fully satisfied with the knowledge of this science obtained in the medical school. The training thus secured became of great practical value during his later career; in fact, made it possible for him to command a wide circle of readers for his "Dental Metallurgy," which reached a fourth edition in 1900.

He was appointed to the position of Demonstrator in the Philadelphia Dental College, and at the close of service of two years in this capacity the honorary degree of Doctor of Dental Surgery was conferred upon him.

Upon the death of Professor E. Wildman in 1876, Dr. Essig was elected to the chair of Mechanical Dentistry and Metallurgy in the Pennsylvania College of Dental Surgery.

Before the completion of two years in this college, there came overtures from the University of Pennsylvania to this faculty to combine the forces of the two institutions into one dental department at the University. This was not acceptable to the majority of the faculty of the Pennsylvania College of Dental Surgery, but Dr. Essig and Dr. E. T. Darby, then professor of Operative Dentistry and Dental Histology, in 1878, arranged with the University authorities to establish a dental school in connection with the Medical Department, and they were accordingly elected on March 15, 1878, to their respective chairs. The department held its first session 1878 and 1879 with Dr. Essig as secretary, he having been elected to that position on March 15 of that year, the deanship not being recognized at that time in the professional schools.

The Department of Dentistry flourished under Dr. Essig's care, but the work trespassed much upon his valuable time, and he resigned the position of secretary in 1883, and James Truman, then a member of the faculty, was elected to fill the position. Subsequently the trustees changed the title of secretary to that of dean, the title given the presiding officer in all departments of the University at the present time.

Dr. Essig continued to perform his duties as Professor of Mechanical Dentistry and Metallurgy up to a few months prior to his death, a period of twenty-three years. On July 5, 1901, he tendered his resignation to the Provost of the University, severing all connection with university work.

It was during this period of twenty-three years that the great development of this school took place, and to Dr. Essig is due a full share of honor for the progress made, for his influence was always on the side of advanced development. He and Professor Darby laid the foundation and successfully tided over the most difficult period of the school's life. That the department received the benefit of his growing reputation, national and international, there can be no question. His lectures on his favorite branch were regarded as superior, exhibiting, as they did, a thorough mastery of the subjects treated.

His earlier work in ceramics in the Hall Laboratory enabled him not only to clearly explain the delicate process of tooth manufacture, but also gave him special skill in what has been known as the "continuous gum" artificial denture, the preparation of which requires ability of a high order.

The first edition of Dr. Essig's work on Dental Metallurgy attracted immediate attention, as it was the first of its kind; indeed, was in advance of the study in the schools, for at that period the subject was not generally taught in the dental colleges. It proved to be an inspiriting force, for very soon it was made part of the curriculum of all the higher schools. The book, therefore, was warmly received, both at home and abroad, and was translated into French, German, and Russian languages, and reached its fourth edition in 1900.

Dr. Essig was selected to edit "The American Text-Book of Prosthetic Dentistry," in the production of which he was ably seconded, as assistant, by the valuable labors of Dr. H. H. Burchard, whose early death has not ceased to be a source of sorrow to his many friends. The cyclopædic form of this book destroyed, to some extent, individuality, but it added materially to a reputation firmly established. This book is now in its second edition, and promises to continue to bear the character stamp of its editor through many future editions.

Dr. Essig was not a prolific writer in periodical literature, nor was he an active participant in either local, State, or national organizations; in fact, his taste did not seem inclined that way, much to the regret of his coworkers, who felt his ability should have had a wider field and been more marked in the higher councils of his profession.

He inherited artistic tastes and was an artist of very considerable ability. He helped to organize the Art Club of Philadelphia, and was an active member of this organization to the day of his death. He was also a member of the Masonic Fraternity.

He possessed a home at Wallingford beautiful in its surroundings and adorned with his excellent taste.

Thus for forty-four years Dr. Essig filled the full measure of professional life. He was one of the men who made dentistry what it is to-day. He entered into it in the early part of the last half of the nineteenth century and lived to see the twentieth open with much that was crude in dentistry laid aside, and much that he so laboriously acquired at the time of his early studies, and so valuable, practically forgotten. The number of those active in the labor of the past century is growing smaller year by year, but the work they did is living, it may be silently, but still living in the skill and knowledge of those who profited by their instruction and their labor. Among these there will not be many whose names will go down on the pages of dental history more honored for his work than Dr. Charles J. Essig.

He leaves a wife and two sons, both of whom have followed in their father's footsteps, both graduating at the Department of Dentistry, University of Pennsylvania.

The funeral took place from his home at Wallingford; interment at South Laurel Hill Cemetery.

